# Supplementary material for: Resolving the full spectrum of human genome variation using Linked-Reads
Source: Genome Res. 2019 Apr;29(4):635–45. doi: 10.1101/gr.234443.118 (PMC6442396; doi:10.1101/gr.234443.118)
Supplement: Supplemental Material [file supp_gr.234443.118_Supplemental_longranger-2.2.zip › longranger-2.2.tar.bz2!/lr2.2-public/lib/stan/cmdstan/stan/doc/api/html/cholesky__decompose_8hpp_source.html]

Stan: src/stan/math/matrix/cholesky\_decompose.hpp Source File


|  |  |
| --- | --- |
|  | Stan  2.5.0  probability, sampling & optimization |


- Main Page
- Related Pages
- Namespaces
- Classes
- Files

- File List
- File Members

All Classes Namespaces Files Functions Variables Typedefs Enumerations Enumerator Friends Macros Pages

- src
- stan
- math
- matrix

cholesky\_decompose.hpp

Go to the documentation of this file.

1 #ifndef STAN\_\_MATH\_\_MATRIX\_\_CHOLESKY\_DECOMPOSE\_HPP

2 #define STAN\_\_MATH\_\_MATRIX\_\_CHOLESKY\_DECOMPOSE\_HPP

3

4 #include <stan/math/matrix/Eigen.hpp>

5 #include <stan/math/error\_handling/matrix/check\_square.hpp>

6 #include <stan/math/error\_handling/matrix/check\_symmetric.hpp>

7

8 namespace stan {

9  namespace math {

10

21  template <typename T>

22  Eigen::Matrix<T,Eigen::Dynamic,Eigen::Dynamic>

23  cholesky\_decompose(const Eigen::Matrix<T,Eigen::Dynamic,Eigen::Dynamic>& m) {

24  stan::math::check\_square("cholesky\_decompose(%1%)",m,"m",(double\*)0);

25  stan::math::check\_symmetric("cholesky\_decompose(%1%)",m,"m",(double\*)0);

26  Eigen::LLT<Eigen::Matrix<T,Eigen::Dynamic,Eigen::Dynamic> >llt(m.rows());

27  llt.compute(m);

28  return llt.matrixL();

29  }

30

31  }

32 }

33 #endif

stan::math::cholesky\_decompose

Eigen::Matrix< T, Eigen::Dynamic, Eigen::Dynamic > cholesky\_decompose(const Eigen::Matrix< T, Eigen::Dynamic, Eigen::Dynamic > &m)

Return the lower-triangular Cholesky factor (i.e., matrix square root) of the specified square...

**Definition:** cholesky\_decompose.hpp:23

check\_symmetric.hpp

stan::math::check\_symmetric

bool check\_symmetric(const char \*function, const Eigen::Matrix< T\_y, Eigen::Dynamic, Eigen::Dynamic > &y, const char \*name, T\_result \*result)

Return true if the specified matrix is symmetric.

**Definition:** check\_symmetric.hpp:31

Eigen.hpp

stan::math::check\_square

bool check\_square(const char \*function, const Eigen::Matrix< T\_y, Eigen::Dynamic, Eigen::Dynamic > &y, const char \*name, T\_result \*result)

Return true if the specified matrix is square.

**Definition:** check\_square.hpp:24

check\_square.hpp


---

[ Stan Home Page ]

*© 2011–2014,
Stan Development Team.*
